# Unsupervised Event Characterization and Detection in Multichannel Signals: An EEG application

**DOI:** 10.3390/s16040590

**Published:** 2016-04-23

**Authors:** Angel Mur, Raquel Dormido, Jesús Vega, Natividad Duro, Sebastian Dormido-Canto

**Affiliations:** 1 Department of Computer Sciences and Automatic Control, UNED, Juan del Rosal 16, 28040 Madrid, Spain; raquel@dia.uned.es (R.D.); nduro@dia.uned.es (N.D.); sebas@dia.uned.es (S.D.-C.); 2 National Fusion Laboratory by Magnetic Confinement, CIEMAT, Complutense 40, 28040 Madrid, Spain; jesus.vega@ciemat.es

**Keywords:** artifacts, EEG, event characterization, event detection, unsupervised classification

## Abstract

In this paper, we propose a new unsupervised method to automatically characterize and detect events in multichannel signals. This method is used to identify artifacts in electroencephalogram (EEG) recordings of brain activity. The proposed algorithm has been evaluated and compared with a supervised method. To this end an example of the performance of the algorithm to detect artifacts is shown. The results show that although both methods obtain similar classification, the proposed method allows detecting events without training data and can also be applied in signals whose events are unknown *a priori*. Furthermore, the proposed method provides an optimal window whereby an optimal detection and characterization of events is found. The detection of events can be applied in real-time.

## 1. Introduction

Electroencephalography (EEG) is a non-invasive method to record electrical activity of the brain using sensors distributed along the scalp. Signals detected by an EEG with no connection to a specific brain activity are called artifacts. These are usually classified as physiological and non-physiological artifacts. Physiological artifacts are generated by the patient and non-physiological artifacts can arise from outside of the body (*i.e.*, equipment, environment, *etc.*).

In many cases the information that is hidden behind the physiological artifacts is relevant to a proper diagnosis. Think, for instance, about early detection of mental fatigue or in monitoring stress levels. Others health applications need EEG signals without artifacts contamination in order to reduce the misinterpretation of the EEG and limit the potential for adverse clinical consequences. In this case brain computer interfaces need to filter artifacts in real time [1,2]. Consequently, it is necessary, especially for long recordings, to develop methods to detect and correctly identify artifacts either for analyzing or removing them. This is the main goal of this paper.

There are some approaches for detecting and removing artifacts using EEG recordings. Statistical methods as SCADS (Statistical Control of Artifacts in Dense Array Studies) [3] and FASTER (Fully Automated Statistical Thresholding for EEG Artifact Rejection) [4] detect and remove artifacts to analyze event-related potentials. The packages EEGLAB [5] and FieldTrip [6] include some routines to detect EEG artifacts but some threshold values need to be defined. Reference [7] is a practical example of how threshold values are used for detection of EEG artifacts in polysomnographic recordings.

DETECT [8] is a toolbox for detecting and identifying artifacts based on the supervised classifier support vector machine (SVM). Note that other supervised methods do not carry out an identification process [9,10,11,12]. An advantage of DETECT over other methods is that the user is not required to manually define threshold values (with the exception of a certainty value applied to the classification outcome to reduce false positives). Moreover it captures the frequency and duration of artifacts. DETECT only finds artifacts included in the training process and the quality of this detection depends on the quality of the training data.

In [13], the authors present a practical example of how a SVM classifier is used to detect artifacts arising from head-movements. Reference [14] describes a novel application of one-class support vector machine novelty detection for detecting seizures in humans.

In this paper we first propose a new, simple, and effective unsupervised method (UMED) to characterize and detect events in multichannel signals. Then the proposed method is applied to detect artifacts in EEG recordings. We use the term “artifact detection” interchangeably with the term “event detection” although artifact is more closely associated with the EEG recordings.

Some advantages of the proposed method are the following: (i) it does not need to manually specify any threshold value; (ii) it allows the measurement of the frequency and duration of the events; (iii) as an unsupervised method it does not need training data; (iv) it can be applied to signals with *a priori* unknown events; (v) it provides an optimal detection technique to characterize the events by finding an optimal window; (vi) it can be used in real-time; and (vii) it provides the number of independent components (NIC) required to remove artifacts by means of an independent component analysis (ICA) [2,15,16].

This paper is organized as follows. Section 2 presents some terms and theoretical background used to implement UMED. The proposed unsupervised method to characterize and detect events in multichannel signals is presented in Section 3. In Section 4, we test this method using an EEG recordin*g* with artifacts. Finally in Section 5 and Section 6, a discussion and conclusions of the paper are respectively presented.

## 2. Background

### 2.1. Events and Multi-Channel Signals

In this section, we formally define the terms “event” and “multichannel signal”. Both are linked to the concept of temporal sequence. Temporal sequence refers to a sequence of happenings or events in a time interval. An event is something that happens at a time *t_E_* and it reflects some kind of change in a temporal evolution. An important characteristic of temporal sequences is the order in which events take place. Another important characteristic is the duration *d_E_* between *t_E_* and the next event.

A temporal sequence is normally represented by a series of nominal symbols from a particular alphabet. Every event is characterized by a symbol. In this way, an event with symbol *E* is described by two elements (*E*, *t_E_*). The three elements (*E*, *t_E_*, *d_E_*) represent a state. A temporal sequence *P* made of *u* events *E^i^* for *i* = 1, …, *u* can be described by a *u*-tuple of states *P* = <(*E*^1^, *t_E_*^1^, *d_E_*^1^), (*E*^2^, *t_E_*^2^, *d_E_*^2^), …, (*E*^u^, *t_E_*^u^, *d_E_*^u^)>.

In general, the number and temporal locations of events can be unknown within a temporal sequence. This means that events have to be recognized and located inside the sequence. It occurs, for example, in the monitoring of a physical system with *Q* sensors for a period of time [*T*_1_, *T*_2_]. In this case, every sensor represents a channel and simultaneously provides a signal describing part of the whole information. Signals of this type that are generated by multiple sensors are called multichannel (MC) signals. In other words, a MC signal refers to a set of signals that show cross-channel similarity or correlation. A MC signal with *Q* channels is represented by a vector *X*(*t*) = [*CH*_1_(*t*) *CH*_2_(*t*), …, *CH_Q_*(*t*)] where *CH_q_*(*t*) is the signal of the channel *q* for *q* = 1, 2, ..., *Q*. A *MC* signal *X*(*t*) with events can also be described, in compact form, as a temporal sequence *P*.

### 2.2. The Hierarchical Clustering, the Cophenetic Correlation Coefficient, and the S_Dbw Validity Index

In this section we briefly describe three concepts used in the implementation of the proposed algorithm: the hierarchical clustering (HC) algorithm [17], the Cophenetic Correlation Coefficient (CCC) [18], and the *S_Dbw* Validity Index [19].

The HC groups data over a variety of scales by creating a cluster tree or dendrogram. It follows this general procedure: (1) find the similarity or dissimilarity between every pair of objects in the data set; (2) group the objects into a binary, hierarchical cluster tree (linkage); (3) determine where to cut the hierarchical tree into clusters.

In a dendrogram, the cophenetic distance between two objects is represented by the height of the link at which those two objects are first joined. That height is the distance between the two subclusters that are merged by that link. The CCC for a cluster tree is defined as the linear correlation coefficient between the cophenetic distances obtained from the tree, and the similarities (or dissimilarities) used to construct the tree. The CCC is a measure of how faithfully a dendrogram maintains the original pairwise distances. The magnitude of CCC should be very close to 1 to achieve a high-quality solution of the HC algorithm.

Given a dataset, if *x*(*O_i_*, *O_j_*) is the distance between the *i*th and *j*th object, *t*(*O_i_*, *O_j_*) is their cophenetic distance, $\bar{x}$ is the average of the *x*(*O_i_*, *O_j_*) and $\bar{t}$ is the average of the *t*(*O_i_*, *O_j_*), then the CCC is given by Equation (1):

(1) $$CCC=\frac{\sum_{i<j}\left(x\left(O_{i},O_{j}\right)-\bar{x}\right)\left(t\left(O_{i},O_{j}\right)-\bar{t}\right)}{{\left(\left[\sum_{i<j}{\left(x\left(O_{i},O_{j}\right)-\bar{x}\right)}^{2}\right]\left[\sum_{i<j}{\left(t\left(O_{i},O_{j}\right)-\bar{t}\right)}^{2}\right]\right)}^{1/2}}$$

The *S_Dbw* validity index is used for measuring “goodness” of a clustering result. Its definition is based on cluster compactness and separation but it also takes into consideration the density of the clusters. Lower index value indicates better clustering schema.

Given a set of *k* clusters *G_i_* (*i* = 1, …, *k*) of the dataset *DS*, the *S_Dbw* index is defined in the following way:

(2) $$S\underline{\;}Dbw\left(k\right)=Scat\left(k\right)+Dens\underline{\;}bw\left(k\right)$$

where *Scat* is the intra-cluster variance that measures the average scattering of clusters and it is described by:

$$Scat\left(k\right)=\frac{1}{k}\sum_{i=1}^{k}\frac{\left\|\sigma \left(G_{i}\right)\right\|}{\left\|\sigma \left(DS\right)\right\|}$$

*σ*(*DS*) is the variance of the dataset *DS* and *σ*(*G_i_*) is the variance of the cluster *G_i_*.

The inter-cluster density *Dens_bw* is defined as follows:

$$Dens\underline{}bw\left(k\right)=\frac{1}{k\left(k-1\right)}\sum_{i=1}^{k}\left(\sum_{j=1,j\neq i}^{k}\frac{density\left(G_{i}UG_{j}\right)}{max\left[density\left(G_{i}\right),density\left(G_{j}\right)\right]}\right)$$

where $density\left(G\right)=\sum_{i=1}^{\left|G\right|}f\left(x_{i},\mu \right),$ *μ* is the center of the cluster *G*, |*G*| is the number of objects in *G*, and the function *f*(*x_i_*, *μ*) is defined by:

$$f\left(x,\mu \right)=\left\{\begin{array}{c} \;0\;if\;distance\left(x,\mu \right)>\frac{1}{k}{\left(\sum_{i=1}^{k}\left\|\sigma \left(G_{i}\right)\right\|\right)}^{1/2}\\ \;1\;otherwise\; \end{array}\right\}$$

If *G* = *G_i_ U* *G_j_*, then *μ* is the middle point of the line segment that is defined by the *μ_i_* and *μ_j_* clusters centers.

## 3. Method for the Characterization and Detection of Events in Multichannel Signals

In this section, we propose an unsupervised method (UMED) to characterize and detect the events of a *MC* signal of duration [*T*_1_, *T*_2_] whose events are a priori unknown. A small temporal window slides along the interval [*T*_1_, *T*_2_]. For each window displacement, it is obtained a feature vector of *S* variables which picks up the behavior of the *MC* signal. A feature vector unsupervised classification is carried out, which allows automatic characterization and detection of the events. The quality of this characterization (and the number of events detected) depends significantly on the size of the sliding window. This is why the algorithm searches for an optimal window size that provides the maximum compactness and separation of the content between the events.

The UMED detects and characterizes events by means of an optimal unsupervised classification. The *S*_*dbw* index and the HC algorithm are used to find the best window along with its unsupervised classification characterized by its optimal number of clusters.

**Figure 1:**
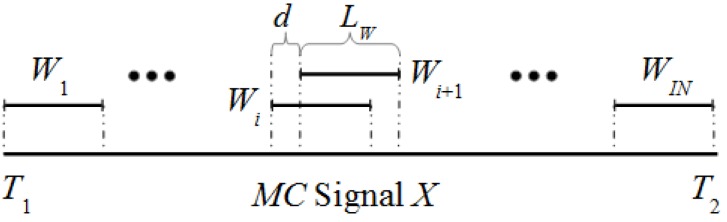
Different windows along the *MC* signal *X*.

Given a *MC* signal *X* for a period of time [*T*_1_, *T*_2_] with unknown events to be characterized and detected, the steps of UMED can be summarized as follows:

**Step 1: Specification of windows.** The algorithm uses a sliding window approach. Figure 1 shows the details of the sliding window in a *MC* signal *X* along the interval [*T*_1_, *T*_2_]. If the window *W_i_* of size *L_w_* is defined by the interval [*t*_1_, *t*_2_] then the window *W_i_*_+1_ is defined by [*t*_1_ + *d*, *t*_2_ + *d*] with *d* > 0. As first step, a set of *IN* windows is selected for a specific *L_w_* and *d*.

**Step 2: Feature matrix (*FV*) calculation.** In this step a matrix *FV* of *IN* rows is generated. Each row *i* of *FV* represents a *Q*-feature vector of the window *W_i_*. So first we calculate for each channel *q* and window *W_i_* a feature vector of size *s*. Then, these features vectors are concatenated to form a unique *Q*-feature vector *Fv_i_* (with *S* = *Q* × *s* features) for each *W_i_*. At last, all the feature vectors *Fv_i_* (for *i* = 1, ..., *IN*) are saved in a matrix *FV* of size *IN* × *S* concatenating them vertically.

Two autoregressive coefficients are good candidates to form the feature vector for each channel *q* and window *W*. The coefficients *a*_1_ and *a*_2_ of an autoregressive model *AR*(2) of a window *W*(*r*) for a single channel can be written as:

(3) $$W\left(r\right)=\sum_{r=1}^{2}a_{r}W\left(r-i\right)+e\left(r\right)$$

where *e*(*r*) is zero-mean white noise. The *a_i_* coefficients can be estimated using Burg’s method [20]. Normally the *Q*-feature vector with a *AR*(2) (of length *Q* × 2) offers superior or equal performance to detect artifacts in EEG recordings than a *AR*(*p*) with *p* > 2 [8,21], and it is computationally less intensive.

**Step 3: Selection of a window size *L_w_* using the CCC.** In this step, first the CCC of the *IN* rows of the matrix *FV* is calculated. If CCC reaches approximately a particular threshold *U*, it is selected the value of *L_w_*. This value is named *L_w_^0^* and its feature matrix *FV^0^*. In case CCC is much lower than *U*, we go back to the step 1 to choose a larger *L_w_*, and then a new *FV* and CCC are tested.

The CCC threshold *U* is defined as 0.85. This value comes from experience and guarantees that the HC algorithm will find a significant classification. From a practical point of view, this threshold is used to reduce the grid search for the window length in step 4. For small window sizes (some dozens of samples) the CCC value can be <0.85. In this case the CCC is used to determine a minimum window size from which the step 4 will start. In step 4, the *S*_*dbw* takes the responsibility to calculate an optimum window size. A low value of *S*_*dbw* comes with a high CCC value. However, the CCC is not defined by compactness and separation of groups and it cannot be used to determine an optimal group number.

For each window size the processing time of CCC is low. So a possible strategy to determine *L_w_^0^* is to select a small group of window sizes and check if the CCC > 0.85 is respected from a particular window size.

**Step 4: Selection of an optimal window size (*L_w_^H^*) and its unsupervised classification.** In this step, first the size of the window is increased *N_L_* times by means of *L_w_^m^* = *L_w_^0^* + *m* × *D_L_* for *m* = 0, ..., *N_L_* and the integer *D_L_* ≥ 1. Second, for each *L_w_^m^*, it is calculated its feature matrix *FV^m^*. Third, the HC algorithm is applied *N_G_* times to classify the rows of each matrix *FV^m^* in different partitions *i.e.*, in 2, 3, ..., *N_G_* + 1 groups. Fourth, for each partition, its *S_Dbw^m^_g_* index (with *g* = 2, ..., *N_G_* + 1) is calculated. The minimum value of the index, named *MIn^m^*, determines the optimal number of groups *No^m^*. The minimum value *MIn^H^* between the *Min’s* determines the optimal window size *L_w_^H^*, the optimal feature matrix *FV^H^* and the optimal number of groups *No^H^*.

The selected *N_L_* value allows to check a significant number of window sizes. The *MC* signal *X* has an initial period of time (IPT) where there are no events. If IPT is known, then *Lw^NL^* has to be <*IPT*. If IPT is not known, then it is possible to add at the start of *X* a synthetic portion of signal without events. When the size *L_w_^H^* is near to *Lw^NL^* a bigger value of *N_L_* is chosen (with the help of a synthetic IPT, if necessary) to ensure that *L_w_^H^* is well surrounded by other suboptimal sizes.

The selected *N_G_* value allows to check a significant number of partitions. In case the optimal number *No^m^* is near to *N_G_*, a bigger value of *N_G_* is chosen to ensure that *No^m^* is well surrounded by other suboptimal group numbers.

**Step 5: Event Detection by means of the optimal unsupervised classification.** It is accomplished the unsupervised classification for the predetermined number of groups *No^H^*. The condition CCC > *U* and the selection of *MIn^H^* allow the HC algorithm to find a high-quality classification with the best group compactness and separation taking into consideration the density of the clusters.

Once all the windows of size *L_w_^H^* have been classified into *No^H^* clusters, the different events can be detected along the *MC* signal *X*. After this classification, we will use, preferably, the term interval instead of the window to stress the window time interval.

The events are characterized by the group numbers of the optimal classification. These are placed at the beginning and the end of each group of consecutive intervals [*W_i_*, *W_i+_*_1_, ..., *W_j_*] where (*1* ≤ *i* ≤ *j* ≤ *IN*) with the same group number. The beginning and the end can be defined using the next formula [8]:

(4) $$\left[\left(i-1\right)\times d+M\times L_{w}-\frac{d}{2},\left(j-1\right)\times d+M\times L_{w}+\frac{d}{2}\right]$$

where *i* is the number of the first intervals *W_i_*, *j* is the number of the last interval *W_j_*, *d* is the slide width, and *M* = 6/7. The product *L_W_* × 6/7 is more accurate than using the midpoint with *M* = 1/2. Equation (4) uses only *d* samples per interval along the signal without overlapping. In this way, intervals between events are uniquely labeled regardless of whether events are placed in the intersection of two overlapping windows classified in a different way. Equation (4) is used to plot the events along the EEG signal. It finds the events’ temporal locations along the EEG signal and, thus, it is possible to study their behavior.

## 4. Testing the Algorithm

### 4.1. EEG multichannel Signal with Artifacts

**Figure 2:**
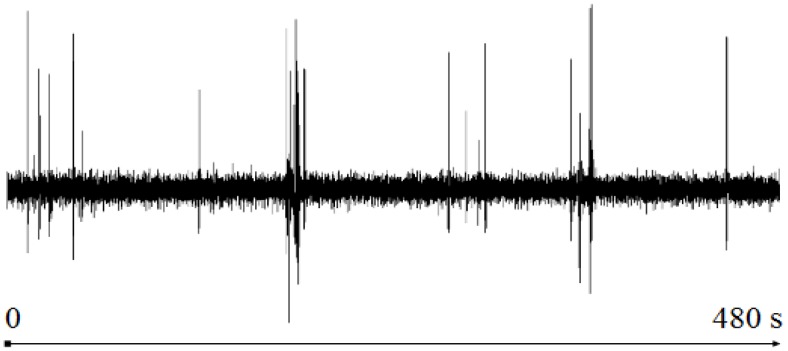
The channel *CH*_1_(*t*) of the 64-channel EEG.

The proposed algorithm has been tested to detect artifacts in multichannel EEG signals. The test signal is an EEG recording of 8 min duration used in [8] to highlight the utility of the DETECT toolbox. The data are sampled at 256 Hz using a 64-channel Biosemi Active Two System (Amsterdam, Netherlands). Figure 2 shows the channel *CH*_1_(*t*) of the EEG recording. Our test uses all of the channels.

The artifacts are categorized into six classes [8]: None (NN), Jaw Clench (JC), Jaw Movement (JM), Eye Blink (EB), Eye Left Movement (ELM), and Eye Up Movement (EUM). In reference [8] a SVM approach is used for detection of artifacts. This supervised method (SM) is trained with a balanced set of 20 trials for each type of artifact along with a window of *L_W_* = 128 samples and a slide width of *d* = 32 samples. Each window of a channel is characterized by the first two autoregressive coefficients [21]. A number of 3837 windows (or intervals) are analyzed and classified. Furthermore, in [8] a certainty thresholding policy is applied to this classification to remove false positives in the data. The Table 1 summarizes the SM outcome after using a value of 0.5 to threshold the certainty.

### 4.2. Detection and Characterization of Events Using EEG Recordings

In this section, we apply the proposed UMED method presented in Section 3 to detect and characterize the artifacts in the EEG recording described above. A comparison with the results summarized in Table 1 is also given. Both approaches can be compared because they use the same AR feature vectors and a similar sliding window approach.

The first two autoregressive coefficients have been used as feature vector per window and channel. For *L_w_* = 128 samples, the CCC is 0.8510 and *No* = 7. The Table 2 shows (for the step 4) the CCC values, the *MIn* values and the optimal number of clusters for different *L_w_* > 128 samples starting at *L_w_* = 135 and *D_L_* = 5.

From the data presented in Table 2 it is easy to find the minimum value of the index (*MIn)* among the minimums, *Min^H^* = 0.77; the optimal number of clusters *No^H^* = 7 for *L_w_^H^* = 155 samples, and CCC*^H^* = 0.8658. Then, an unsupervised classification (HC algorithm), as stated in step 5, allows finding of the optimal content of the seven groups.

We use a principal component analysis (PCA) [22] to display the clusters found on a 2D representation. PCA is a projection method that re-expresses a collection of correlated variables into a smaller number of variables called principal components, which maximizes the variance of the data.

If the original data formed by *n_v_* vectors of *l* features are stored in a matrix *O_D_*(*l* × *n_v_*), then the PCA transform is:

(5) $$T_{D}=A\left(O_{D}-m_{OD}\right)$$

where *m_OD_* is the mean of the original data and *A* is a matrix whose rows are the eigenvectors of the covariance matrix *C_OD_* = (1/*n_v_*)*O_D_ O_D_*^T^.

Our PCA is carried out using the Equation (5) and *O_D_* = (*FV^H^*)*^T^*. Then, it is selected the submatrix *T_D_*^2^ made up of the first two principal components (the first two rows of *T_D_*). The column *k* of *T_D_*^2^ provides a new feature vector of only two elements to the window *W_k_*.

**Figure 3:**
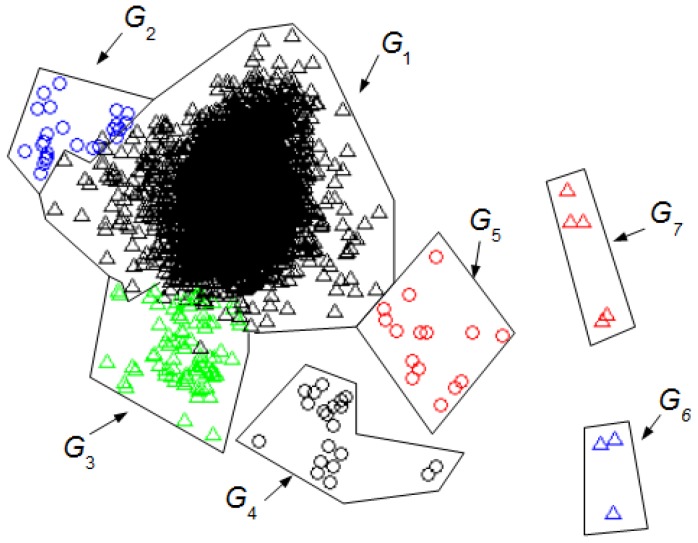
Groups of intervals found using the unsupervised classification (UMED) and a PCA. The *L_w_^H^* has 155 samples. The first two principal components contain 55% of the full information. The different clusters are characterized but not identified.

Figure 3 shows the seven optimal clusters found. The interval *z* is represented by a point whose coordinates are *T_D_*^2^(1, *z*) and *T_D_*^2^(2, *z*). Each cluster is distinguished by a specific shape and color. Table 3 summarizes the UMED outcome for *L_w_^H^* = 155.

**Figure 4:**
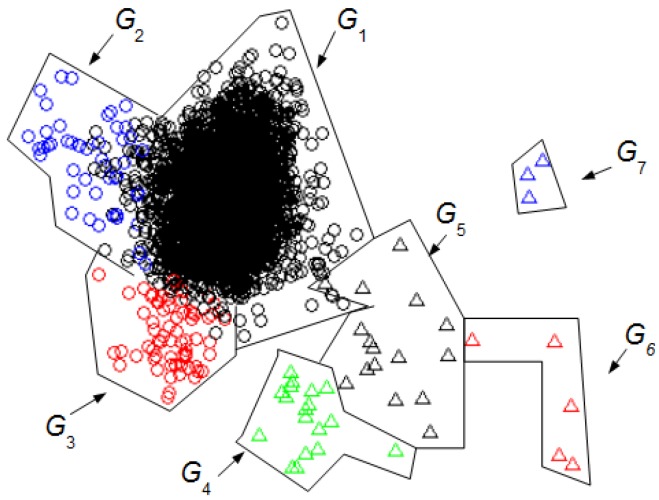
Groups of intervals found using the unsupervised classification (UMED) and a PCA. The *L_w_* has 128 samples. The first two principal components contain 53% of the full information. The different clusters are characterized but not identified.

Figure 4 shows the seven clusters found using our UMED for *L_w_* = 128. Table 4 summarizes the UMED outcome. This result is important to compare UMED with SM.

**Figure 5:**
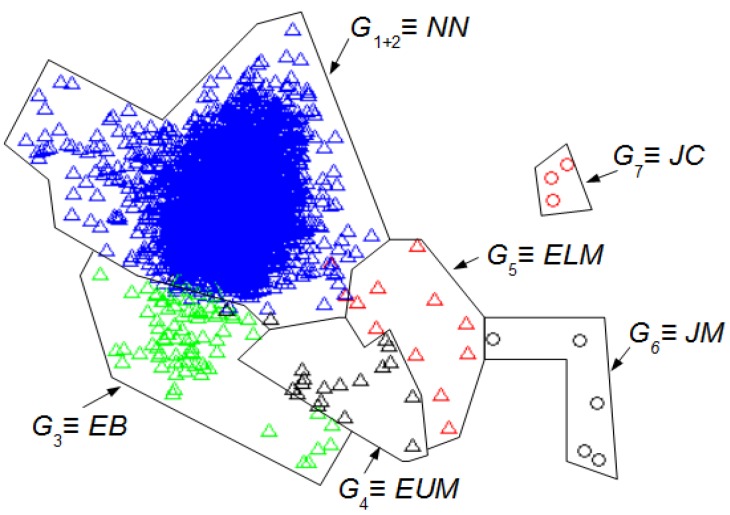
Groups of intervals found using the supervised classification (SM) [8] and the first two principal components of the Figure 4. The *L_w_* has 128 samples. The first two principal components contain 53% of the full information. The different clusters are identified. Using the Table 1: NN intervals are in *G*_1+2_, the EB in *G*_3_, the EUM in *G*_4_, the ELM in *G*_5_, the JM in *G*_6_, and the JC in *G*_7_.

Figure 5 shows the 6 clusters found with DETECT [8] using a supervised classification (SM) for *L_w_* = 128 and a value of 0.5 to threshold the certainty. The principal components are the same as those of Figure 4. Table 1 summarizes the SM outcome where the NN intervals are in *G*_1+2_, the *EB* in *G*_3_, the EUM in *G*_4_, the ELM in *G*_5_, the JM in *G*_6_, and the JC in *G*_7_.

Figure 4 and Figure 5 allow the comparison of the UMED and the SM. The UMED finds a group *G*_2_ that is not detected when using SM because the intervals of this group do not form part of the required training process in the SM. Consequently, these intervals are integrated into the *G*_1+2_ group that contain the NN intervals. It is observed that SM obtains groups more blurred than the UMED but, in general, the detection of intervals is similar. The only exception are around seven intervals between group *G*_3_ and *G*_4_, and around six intervals between groups *G*_4_ and *G*_5_. Moreover, the Normalized Mutual Information (NMI) index [23] allows to compare the intervals’ group numbers for both methods. If, for the classification of UMED, the *G*_2_ is included into the *G*_1_, the NMI between the UMED and SM result is 0.8317. This score is highly consistent since UMED and SM find a similar classification of intervals. A value of NMI close to 1 means that the results of both methods are similar.

**Figure 6:**
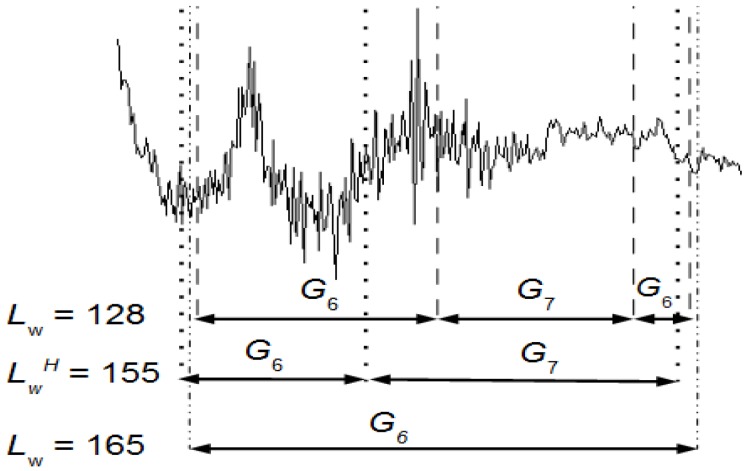
Events detected between the samples 46,389 and 46,653 using a *L_w_^H^* = 155, *L_w_* = 128 and *L_w_* = 165. The signal is a portion of an EEG channel.

The groups *G*_6_ and *G*_7_ have events that are consecutive in time. Figure 6 shows the effect on the classification of these groups using a *L_w_^H^* = 155, *L_w_* = 128 and *L_w_* = 165 (see also Table 2). By using Equation (4) the events can be temporally located along the EEG signal and, thus, it is possible to study their behavior.

The optimal *L_w_^H^* provides two groups *G*_6_ (intervals 1447, 1448, 1449) and *G*_7_ (intervals 1450, 1451, 1452, 1453, 1454) well separated and these groups are mathematically more significant than the *L_w_* = 128 ones (intervals 1448, 1449, 1450, 1451, 1455 for *G*_6_ and 1452, 1453, 1454 for *G*_7_). The *G*_6_ of *L_w_^H^* has one artifact and the one of *L_w_* = 128 two artifacts. The *L_w_* = 165 yields one group *G*_6_ and this is why, in Table 2, its *No* is 6.

Figure 6 shows, graphically, how the quality of the results depends on the selected sliding window size.

## 5. Discussion

Artifacts can be either analyzed or filtered from an EEG recording. The UMED, as the SM approach, has been mainly developed to analyze artifacts, to study for instance the frequency and the duration of artifacts. The UMED also provides, in a unsupervised way, the number of different artifacts. For instance, in Figure 6 and for *L_w_^H^*, the number of artifacts is 2: JM and JC. The durations of JM and JC are 0.37 s and 0.62 s, respectively. Both appear once during the entire EEG recording.

In Section 4 the proposed UMED has been evaluated by comparing its classification result with the one obtained with the SM using the same *L_w_*, *d* and EEG recording. It has been used with the PCA and the NMI to contrast, visually and quantitatively, both classifications. This evaluation is significant because, in addition to the fact that two different methods UMED and SM obtain similar results, the SM method was validated comparing “user labelings” with “automated labelings”. The SM method was validated by measuring the agreement (in seconds) between expert and SM labeling in some EEG recordings. This agreement is, in the worst case, over 80%. The best case reaches about 98%. In this way, we have checked that UMED with the same *L_w_*, *d*, and EEG recording provides a similar result to the one given by an expert. Therefore, UMED could also be considered as an unsupervised expert system.

Unlike SM, UMED determines an optimal window size *L_w_^H^* which allows an optimal classification based on the *S_Dbw* index. With this index and the HC algorithm, UMED characterizes clusters that have different density and can provided new clusters that SM could not have considered during the training process. The HC computes the distance between two data points and the distance between two clusters (linkage) using, respectively, the Euclidean and the average distance (the average distance between each element in one cluster to every element in the other cluster).

The quality of the SM outcome depends on the quality of the training process but, as UMED, it also depends on the size of the sliding window selected. As said in [8]: “If the window size is too small, then the window may not contain sufficient information to model the feature. On the other hand, if the window is too large, SM will not be able to distinguish closely-spaced events, and temporal precision regarding the exact onset of an event is diminished”. This is also true for UMED. Unlike UMED, SM has no mathematical criteria to decide the sliding window size, except to check different sizes and compare the results with the opinion of an expert. This process seems slower and more tedious than the one of UMED.

We can compare the Figure 3 and Figure 4 with Figure 5 to identify the clusters obtained in Figure 3 and Figure 4. In this case, it is not necessary for an expert to identify the clusters found using UMED. In Figure 4, by means of UMED, the groups *G*_3_ and *G*_4_ are clearly distinguished. On the contrary, in Figure 5, SM does not differentiate well these groups because some Eye Up Movement (EUM) are considered as Eye Blink (EB). Some EUM do not follow the PCA representation (the lowest green triangles are in *G*_3_ instead of *G*_4_). Possibly, a better quality of the trials (used for the training process) would have improved the quality of the SM result. In Figure 3, due to the use of *L_w_^H^*, the compactness and separation of the clusters are better than in Figure 4 and Figure 5. Likewise, in Figure 6, the intervals of Jaw Movement *G*_6_ and Jaw Clench *G*_7_ for *L_w_^H^* are better defined.

The SM can be used in real time to detect artifacts in an EEG recording. The classifier has been trained with a balanced set of trials for each type of artifact. Both, the number of artifacts and their trials have to be selected by an expert. Their size *L_w_* is the same for all. The SM classifier uses a sliding window with the same size *L_w_*. In real time, each new interval is classified during the acquisition of the next interval.

The UMED can also work in real time primarily in two ways. The first one (UMED_1_) acts similarly to SM but now the training process of the SVM is different. The UMED automatically detects the number of artifacts and their trials from a significant EEG recording using an optimal window size *L_w_^H^*. Then, an expert only needs to identify one portion of consecutive intervals of each cluster and select a significant number of intervals per cluster. This number has to be adequate to get a balanced set of trials. Once the SVM has been trained with these trials (the result of UMED), it is ready to work in real-time. Similarly to SM, each new interval (from other EEG recording) is classified during the acquisition of the next interval.

The second way (UMED_2_) consists on using UMED as a supervised method without using a SVM. In this case the first step is to get a reference classification from a significant EEG recording similarly to UMED_1_. Unlike UMED_1_ that selects a set of intervals for a training process, UMED_2_ characterizes a dataset *DS*_2_ using the feature vectors of all the intervals (of size *L_w_^H^*). The UMED_2_ selects *DS*_2_ along with its optimal number of clusters *No^H^* and its *S_Dbw* index named *IndN*_o_. An expert only needs to identify one portion of consecutive intervals of each cluster.

Then, UMED_2_ is ready to classify new sets of *Nint* intervals from other EEG recording. Each set of intervals is added to *DS*_2_. The optimal number of clusters is calculated using the *S_Dbw* index and then it is compared the *S_Dbw* index for *No^H^* (named *IndN*_o_) and the one for *No^H^* + 1 (named *IndN*_1_). If the value *IndN_o_* < *IndN*_1_ the solution is the unsupervised classification using HC for *No^H^* groups. If *IndN_o_* > *IndN*_1_ it is compared *IndN*_1_ with *IndN*_2_ and so on until *IndN_i_* < *IndN_i_*_+1_. In general, the solution is the classification using HC for *No_i_^H^* groups when *IndN_i_* < *IndN_i_*_+1_.

Usually with a significant *DS*_2_ (containing all the types of artifacts) each of the *Nint* intervals belongs to one of the *No^H^* groups and *IndN_o_* < *IndN*_1_. The *Nint* intervals are classified at the same time and, after that, their feature vectors are removed from the reference dataset. However, if any of the *Nint* intervals are classified in a new cluster, then it can remain in the dataset. The new cluster will be a new type of artifact, but it is not identified. This is similar to a SVM that only identifies artifacts included in the training process. In real-time, every *Nint* intervals are classified during the acquisition of the next *Nint* intervals.

The SM method identifies the artifacts. The UMED method characterizes and detects the artifacts. The identification process is also possible by means of UMED_1_ and UMED_2_. Thanks to UMED the training process in UMED_1_ and UMED_2_ is simpler and faster than the one of SM. It is understood that UMED_1_ and UMED_2_ can also be used for offline applications.

The UMED does not reduce the feature vectors dimensionality by using for example PCA. The PCA is only used to visually contrast the SM and UMED outcomes. In addition to the loss of information, the use of PCA would be a disadvantage for UMED_1_ and UMED_2_.

Some real-time EEG applications use a small number of electrodes. The use of UMED is not limited by the number of channels. With a lower number, the computational effort decreases, though the quality of the result can change. Usually, with fewer channels, discrimination between artifacts decreases so different artifacts could be classified into the same group. If the goal is the correct identification of the artifacts, then, this rule applies: the more channels, the better the discrimination of artifacts.

Artifacts are superimposed on the real EEG signal. Once they have been detected, they can be directly removed by cutting the segments of the signal where there are overlaps. In this case there is a loss of information because those segments include a part of the real EEG signal. The UMED (that detects artifacts and the portions of signals without artifacts (PWA)), can also be used along with other algorithms, such as ICA [15,16], specialized to filter artifacts leaving the full EEG signal with useful information. The PWA can lose information after using ICA. By replacing the PWA after using ICA with the original PWA detected using UMED ensures the maximum of useful information along with the EEG signal without artifacts. UMED also provides the *NIC* used in an ICA that corresponds to the number of clusters. For the full EEG signal of Figure 1, *NIC* = 7. For a specific EEG segment, this number will depend on the artifacts detected by UMED in that portion of signal. In Figure 6, *NIC* = 3.

There is a large number of recent research papers that present artifact de-noising of EEG signals. Reference [24] presents an algorithm for removing peak and spike noise from EEG. This is based on filtering and thresholding the analytic signal envelope. Reference [25] presents an unsupervised algorithm that uses modified multiscale sample entropy and Kurtosis to automatically identify the independent eye blink artefactual components and, subsequently, denoise these components using biorthogonal wavelet decomposition. This method neither requires manual identification for artefactual components nor an additional electrooculographic channel. The method *FORCe* [26] is an artifact removal method developed for use in brain-computer interfacing. It is based upon a combination of wavelet decomposition, independent component analysis, and thresholding. The method LAMIC [27] does not outperform *FORCe* but it is interesting because the artifact removal from the EEG has been performed by means of a clustering algorithm. The EEMD-ICA approach [28] removes artifacts preserving more information than other classical ICA methods.

The UMED has been used to study physiological artifacts without the presence of electrical interferences. Modern acquisition systems allow for reducing that kind of interference. However, for a specific application, it could be interesting to check the UMED behavior in the presence of non-physiological artifacts. Usually, the UMED will create new clusters if the electrical artifacts are strong and located in specific portions of the signal. In case the electrical interference is weak along the full EEG signal, there would be no new clusters related to that non-physiological artifact and the UMED performance would not change. When the electrical interferences are not the subject of study, the best choice is to avoid them or filter them during the acquisition process. That is imperative when the interference is strong and distributed continuously along the full EEG signal

The UMED method has been presented using an EEG recording. The artifacts are very well-differentiated from the cognitive EEG part without artifacts. This cognitive part is only related to the thinking and its high variability is concentrated in a unique cluster that is the biggest. This cluster is not affected using different subjects since the *AR* coefficients are scale- and location-invariant [8,21]. Normally, there is also a unique cluster per artifact from multiple subjects. It may happen that a type of artifact, from a subject with very different morphology, could be grouped in a different cluster. In this case, we could find more than a cluster per artifact, although the distinction between the different artifacts remains. This possibility can diminish as the number of subjects increases.

The *AR*(2) coefficients are good candidates as feature vectors because they allow differentiation between artifacts in the EEG signal and are also scale- and location-invariant. Furthermore, with *AR*(2), the cognitive part without artifacts is well differentiated and is grouped in a single cluster.

The *AR* coefficients are useful to detect artifacts but they cannot detect cognitive events. In theory, once an EEG recording has been cleaned of artifacts, the UMED could be used along with other type of feature vectors that contain spatio-temporal information to detect cognitive events. The UMED outcome for these feature vectors could be evaluated by using a non-invasive optical technique as Near-infrared spectroscopy (NIRS) [29,30,31,32].

## 6. Conclusions

In this paper a new unsupervised method of classification of events in multichannel signals from a sensor system has been presented (UMED). This classification allows characterization and detection of the events. In particular, UMED has been applied to detect artifacts in EEG *MC* signals. It has also been evaluated and compared with a supervised method (SM) to detect artifacts.

The UMED allows to find an optimal classification of the events. This classification can be applied in real-time, either training a SVM classifier or operating as a supervised method. The UMED has been mainly developed to analyze artifacts. For example, to capture the frequency and duration of artifacts for psycho-physiological studies. The UMED also provides, in an unsupervised way, the *NIC* required to remove artifacts with the help of ICA.

## Figures and Tables

**Table 1:** Number of intervals per group using the SM. The groups are None (NN), Jaw Clench (JC), Jaw Movement (JM), Eye Blink (EB), Eye Left Movement (ELM), and Eye Up Movement (EUM).

Group	Number of Intervals
NN	3715
ELM	12
EUM	21
EB	81
JM	5
JC	3

**Table 2:** Characteristic parameters for the selection of the optimal window (step 4): The window size *L_w_*, the optimal number of clusters *No*, the Cophenetic Correlation Coefficient CCC, and the minimum value of the *S_Dbw* index *MIn*.

*L_w_*	135	140	145	150	155	160	165
CCC	0.8413	0.8564	0.8473	0.8475	0.8658	0.8616	0.8679
*No*	7	7	7	7	7	7	6
*MIn*	0.97	0.80	0.85	0.90	0.77	0.82	1.05

**Table 3:** Number of intervals per group using the UMED with *L_w_^H^* = 155 samples.

Group	Number of Intervals
*G*_1_	3691
*G*_2_	24
*G*_3_	77
*G*_4_	20
*G*_5_	15
*G*_6_	3
*G*_7_	5

**Table 4:** Number of intervals per group using the UMED with *L_w_* = 128 samples.

Group	Number of Intervals
*G*_1_	3680
*G*_2_	46
*G*_3_	67
*G*_4_	18
*G*_5_	16
*G*_6_	5
*G*_7_	3
